# Supplementary material for: Erasable labeling of neuronal activity using a reversible calcium marker
Source: eLife. 2020 Sep 15;9:e57249. doi: 10.7554/eLife.57249 (PMC7492087; doi:10.7554/eLife.57249)
Supplement: Source code 1. [file elife-57249-code1.docx.zip › SourceCode.docx]

*Image analysis and macro scripts for rsCaMPARI photoswitching*

Macro scripts in Fiji software^1^ were used to analyze fluorescence intensities from imaging experiments. For dissociated neuron experiments, channels corresponding to rsCaMPARI green, mRuby3 red, and ChrimsonR-HaloTag(JF635) far-red were deinterleaved. ROIs were either manually drawn around neuronal somatas or automatically selected using the Trainable Weka Segmentation plugin^2^ using a classifier trained on segmenting neuronal somatas. Images were rolling ball background subtracted^3^ and mean pixel values within ROIs were saved in Excel. For zebrafish experiments, a script was generated to calculate cosine similarity between image stacks. Code of the Fiji macro scripts used are reproduced below.

//This is a macro for analysis of rsCaMPARI two channel images from field stimulation

//assays acquired on a Nikon widefield microscope.

//It separates channels, selects ROIs, subtracts background, measures intensities,

//and saves the images.

setBatchMode(false);

topdir = getDirectory("select top directory of dataset");

rawdir = topdir + "raw/";

savedir = topdir + "process/";

file_list = getFileList(rawdir);

var filename_array = newArray(0);

var donkey = newArray(1);

for (i=0; i<file_list.length; i++) {

showProgress(i, file_list.length);

run("Bio-Formats Importer", "open=" + rawdir + file_list[i] + " color_mode=Default rois_import=[ROI manager] view=Hyperstack stack_order=XYCZT");

filename=getTitle();

donkey[0] = filename;

filename_array = Array.concat(filename_array,donkey[0]);

run("Make Substack...", "channels=1 slices=2-201,216-415,430-629");

saveAs("tiff", savedir + filename + "_green_stack");

rename("green_stack");

selectWindow(filename);

run("Make Substack...", "channels=2 slices=1");

saveAs("tiff", savedir + filename + "_red");

rename("pre_red");

selectWindow("pre_red");

run("Trainable Weka Segmentation");

wait(3000);

selectWindow("Trainable Weka Segmentation v3.2.32");

call("trainableSegmentation.Weka_Segmentation.loadClassifier", topdir + "classifier_v2.model");

call("trainableSegmentation.Weka_Segmentation.getResult");

while (!isOpen("Classified image")) {

wait(100);

}

selectWindow("Classified image");

saveAs("tiff", savedir + filename + "_classified");

run("8-bit");

setThreshold(0, 120);

run("Convert to Mask");

run("Watershed");

run("Analyze Particles...", "size=50 exclude add");

roiManager("Save", savedir + filename + "_RoiSet.zip");

selectWindow("green_stack");

run("Subtract Background...", "rolling=50");

roiManager("Multi Measure");

selectWindow("pre_red");

run("Subtract Background...", "rolling=50");

roiManager("multi-measure measure_all one append");

saveAs("Results", savedir + filename + "_intensities.csv");

run("Close All");

selectWindow("ROI Manager");

run("Close");

selectWindow("Results");

run("Close");

}

selectWindow("Log");

run("Close");

//This is a macro for analysis of rsCaMPARI two channel images from spontaneous

//recovery assay acquired on a Nikon widefield microscope.

//It separates channels, selects ROIs, subtracts background, measures intensities,

//and saves the images.

setBatchMode(false);

topdir = getDirectory("select top directory of dataset");

rawdir = topdir + "raw_thermal/";

savedir = topdir + "process_thermal/";

file_list = getFileList(rawdir);

var filename_array = newArray(0);

var donkey = newArray(1);

for (i=0; i<file_list.length; i++) {

showProgress(i, file_list.length);

run("Bio-Formats Importer", "open=" + rawdir + file_list[i] + " color_mode=Default rois_import=[ROI manager] view=Hyperstack stack_order=XYCZT");

filename=getTitle();

donkey[0] = filename;

filename_array = Array.concat(filename_array,donkey[0]);

run("Make Substack...", "channels=1 slices=204,206,208,210,212,214,216,218,220,222,224,226,228,230");

run("Set Label...", "label=T0");

run("Next Slice [>]");

run("Set Label...", "label=T1");

run("Next Slice [>]");

run("Set Label...", "label=T2");

run("Next Slice [>]");

run("Set Label...", "label=T3");

run("Next Slice [>]");

run("Set Label...", "label=T4");

run("Next Slice [>]");

run("Set Label...", "label=T5");

run("Next Slice [>]");

run("Set Label...", "label=T6");

run("Next Slice [>]");

run("Set Label...", "label=T7");

run("Next Slice [>]");

run("Set Label...", "label=T8");

run("Next Slice [>]");

run("Set Label...", "label=T9");

run("Next Slice [>]");

run("Set Label...", "label=T10");

run("Next Slice [>]");

run("Set Label...", "label=T11");

run("Next Slice [>]");

run("Set Label...", "label=T12");

run("Next Slice [>]");

run("Set Label...", "label=reset");

saveAs("tiff", savedir + filename + "_thermal_stack");

rename("thermal_stack");

selectWindow(filename);

run("Make Substack...", "channels=2 slices=1");

saveAs("tiff", savedir + filename + "_pre_red");

rename("pre_red");

selectWindow(filename);

run("Make Substack...", "channels=1 slices=2");

saveAs("tiff", savedir + filename + "_pre_green");

rename("pre_green");

selectWindow("pre_red");

run("Trainable Weka Segmentation");

wait(3000);

selectWindow("Trainable Weka Segmentation v3.2.28");

call("trainableSegmentation.Weka_Segmentation.loadClassifier", topdir + "classifier_v2.model");

call("trainableSegmentation.Weka_Segmentation.getResult");

while (!isOpen("Classified image")) {

wait(100);

}

selectWindow("Classified image");

saveAs("tiff", savedir + filename + "_classified");

run("8-bit");

setThreshold(0, 120);

run("Convert to Mask");

run("Watershed");

run("Analyze Particles...", "size=50 exclude add");

roiManager("Save", savedir + filename + "_RoiSet.zip");

selectWindow("thermal_stack");

run("Subtract Background...", "rolling=50");

roiManager("Multi Measure");

selectWindow("pre_red");

run("Subtract Background...", "rolling=50");

roiManager("multi-measure measure_all one append");

saveAs("Results", savedir + filename + "_intensities.csv");

run("Close All");

selectWindow("ROI Manager");

run("Close");

selectWindow("Results");

run("Close");

}

Array.print(filename_array);

for (i=0; i<file_list.length; i++) {

setResult("Label", i, filename_array[i]);

updateResults();

}

selectWindow("Results");

saveAs("Text", savedir + "mean_redgreenratio_within_mask");

run("Close");

selectWindow("Log");

run("Close");

//This is a macro for analysis of rsCaMPARI three channel images from channelrhodopsin

//assays acquired on a Nikon widefield microscope.

//It separates channels, creates composite images, subtracts background, measures

//intensities, and saves the images.

//Images are manually segmented based on the first red image and the ROIs are saved.

//ROIs that have ChrimsonR value > 10 are considered ChrimsonR positive.

//ROIs that have ChrimsonR value < 4 are considered ChrimsonR negative.

setBatchMode(false);

topdir = getDirectory("select top directory of dataset");

rawdir = topdir + "raw/";

savedir = topdir + "process/";

file_list = getFileList(rawdir);

for (i=0; i<file_list.length; i += 2) {

showProgress(i, file_list.length);

run("Bio-Formats Importer", "open=" + rawdir + file_list[i] + " color_mode=Default view=[Standard ImageJ] stack_order=Default");

filename=getTitle();

selectWindow(filename);

run("Make Substack...", " slices=2,299,453,454,458,755,909,910,914,1211,1365,1366");

run("Set Label...", "label=C1_pre_green");

run("Next Slice [>]");

run("Set Label...", "label=C1_post_green");

run("Next Slice [>]");

run("Set Label...", "label=C1_red");

run("Next Slice [>]");

run("Set Label...", "label=C1_ChrimsonR");

run("Next Slice [>]");

run("Set Label...", "label=C2_pre_green");

run("Next Slice [>]");

run("Set Label...", "label=C2_post_green");

run("Next Slice [>]");

run("Set Label...", "label=C2_red");

run("Next Slice [>]");

run("Set Label...", "label=C2_ChrimsonR");

run("Next Slice [>]");

run("Set Label...", "label=C3_pre_green");

run("Next Slice [>]");

run("Set Label...", "label=C3_post_green");

run("Next Slice [>]");

run("Set Label...", "label=C3_red");

run("Next Slice [>]");

run("Set Label...", "label=C3_ChrimsonR");

saveAs("tiff", savedir + filename + "_cleanup");

run("Stack to Images");

selectWindow("C1_red");

saveAs("tiff", savedir + filename + "_C1_red");

rename("C1_red");

selectWindow("C1_ChrimsonR");

saveAs("tiff", savedir + filename + "_C1_ChrimsonR");

rename("C1_ChrimsonR");

selectWindow("C1_pre_green");

saveAs("tiff", savedir + filename + "_C1_pre_green");

rename("C1_pre_green");

selectWindow("C1_post_green");

saveAs("tiff", savedir + filename + "_C1_post_green");

rename("C1_post_green");

selectWindow("C2_red");

saveAs("tiff", savedir + filename + "_C2_red");

rename("C2_red");

selectWindow("C2_ChrimsonR");

saveAs("tiff", savedir + filename + "_C2_ChrimsonR");

run("Enhance Contrast", "saturated=0.01");

run("RGB Color");

saveAs("tiff", savedir + filename + "_RGB_C2_ChrimsonR");

rename("C2_ChrimsonR");

selectWindow("C2_pre_green");

saveAs("tiff", savedir + filename + "_C2_pre_green");

rename("C2_pre_green");

selectWindow("C2_post_green");

saveAs("tiff", savedir + filename + "_C2_post_green");

rename("C2_post_green");

selectWindow("C3_red");

saveAs("tiff", savedir + filename + "_C3_red");

rename("C3_red");

selectWindow("C3_ChrimsonR");

saveAs("tiff", savedir + filename + "_C3_ChrimsonR");

rename("C3_ChrimsonR");

selectWindow("C3_pre_green");

saveAs("tiff", savedir + filename + "_C3_pre_green");

rename("C3_pre_green");

selectWindow("C3_post_green");

saveAs("tiff", savedir + filename + "_C3_post_green");

rename("C3_post_green");

run("Merge Channels...", "c2=C1_pre_green c6=C1_red create keep");

selectWindow("Composite");

setMinAndMax(350, 2000);

run("Next Slice [>]");

setMinAndMax(350, 9000);

saveAs("tiff", savedir + filename + "_composite_C1_pre");

run("RGB Color");

saveAs("tiff", savedir + filename + "_composite_RGB_C1_pre");

close();

close();

run("Merge Channels...", "c2=C1_post_green c6=C1_red create keep");

selectWindow("Composite");

setMinAndMax(350, 2000);

run("Next Slice [>]");

setMinAndMax(350, 9000);

saveAs("tiff", savedir + filename + "_composite_C1_post");

run("RGB Color");

saveAs("tiff", savedir + filename + "_composite_RGB_C1_post");

close();

close();

run("Merge Channels...", "c2=C2_pre_green c6=C2_red create keep");

selectWindow("Composite");

setMinAndMax(350, 2000);

run("Next Slice [>]");

setMinAndMax(350, 9000);

saveAs("tiff", savedir + filename + "_composite_C2_pre");

run("RGB Color");

saveAs("tiff", savedir + filename + "_composite_RGB_C2_pre");

close();

close();

run("Merge Channels...", "c2=C2_post_green c6=C2_red create keep");

selectWindow("Composite");

setMinAndMax(350, 2000);

run("Next Slice [>]");

setMinAndMax(350, 9000);

saveAs("tiff", savedir + filename + "_composite_C2_post");

run("RGB Color");

saveAs("tiff", savedir + filename + "_composite_RGB_C2_post");

close();

close();

run("Merge Channels...", "c2=C3_pre_green c6=C3_red create keep");

selectWindow("Composite");

setMinAndMax(350, 2000);

run("Next Slice [>]");

setMinAndMax(350, 9000);

saveAs("tiff", savedir + filename + "_composite_C3_pre");

run("RGB Color");

saveAs("tiff", savedir + filename + "_composite_RGB_C3_pre");

close();

close();

run("Merge Channels...", "c2=C3_post_green c6=C3_red create keep");

selectWindow("Composite");

setMinAndMax(350, 2000);

run("Next Slice [>]");

setMinAndMax(350, 9000);

saveAs("tiff", savedir + filename + "_composite_C3_post");

run("RGB Color");

saveAs("tiff", savedir + filename + "_composite_RGB_C3_post");

close();

close();

roiManager("Open", rawdir + file_list[i+1]);

selectWindow("C1_ChrimsonR");

roiManager("Multi Measure");

selectWindow("C1_red");

run("Subtract Background...", "rolling=50");

roiManager("multi-measure measure_all one append");

selectWindow("C1_pre_green");

run("Subtract Background...", "rolling=50");

roiManager("multi-measure measure_all one append");

selectWindow("C1_post_green");

run("Subtract Background...", "rolling=50");

roiManager("multi-measure measure_all one append");

saveAs("Results", savedir + filename + "_intensities_C1.csv");

selectWindow("C1_ChrimsonR");

roiManager("Multi Measure");

selectWindow("C2_red");

run("Subtract Background...", "rolling=50");

roiManager("multi-measure measure_all one append");

selectWindow("C2_pre_green");

run("Subtract Background...", "rolling=50");

roiManager("multi-measure measure_all one append");

selectWindow("C2_post_green");

run("Subtract Background...", "rolling=50");

roiManager("multi-measure measure_all one append");

saveAs("Results", savedir + filename + "_intensities_C2.csv");

selectWindow("C1_ChrimsonR");

roiManager("Multi Measure");

selectWindow("C3_red");

run("Subtract Background...", "rolling=50");

roiManager("multi-measure measure_all one append");

selectWindow("C3_pre_green");

run("Subtract Background...", "rolling=50");

roiManager("multi-measure measure_all one append");

selectWindow("C3_post_green");

run("Subtract Background...", "rolling=50");

roiManager("multi-measure measure_all one append");

saveAs("Results", savedir + filename + "_intensities_C3.csv");

selectWindow("ROI Manager");

run("Close");

selectWindow("Results");

run("Close");

run("Close All");

}

//This is a macro for computation of pixel-based cosine similarity between image

//stacks.

setBatchMode(true);

topdir = getDirectory("select top directory of dataset");

rawdir = topdir + "raw/";

savedir = topdir + "process/";

file_list = getFileList(rawdir);

setOption("ExpandableArrays", true);

root_squared_sums = newArray;

norm_cross_coeff = newArray;

for (i=1; i<=file_list.length; i += 1) {

showProgress(i, file_list.length);

run("Bio-Formats Importer", "open=" + rawdir + file_list[i-1] + " color_mode=Default rois_import=[ROI manager] view=Hyperstack stack_order=XYCZT");

rename("C" + i);

//Calculate summed squares

imageCalculator("Multiply create 32-bit stack", "C" + i,"C" + i);

selectWindow("Result of C" + i);

run("Measure Stack...");

myArray = newArray(nResults);

for ( j=0; j<nResults; j++ ) {

myArray[j] = getResult("RawIntDen", j);

}

NoNaN = Array.deleteValue(myArray, NaN);

total = 0;

for (k=0; k<lengthOf(NoNaN); k++) {

total=total+NoNaN[k];

}

root_squared_sums[i-1] = sqrt(total);

Array.show(root_squared_sums);

close("Result of C" + i);

close("Results");

}

//compute cross-correlation and then normalize by the root sum of squares calculated previously

i=1;

for (j=1; j<=file_list.length; j += 1) {

for(k=1; k<=file_list.length; k += 1) {

imageCalculator("Multiply create 32-bit stack", "C" + j,"C" + k);

selectWindow("Result of C" + j);

run("Measure Stack...");

myArray = newArray(nResults);

for ( l=0; l<nResults; l++ ) {

myArray[l] = getResult("RawIntDen", l);

}

NoNaN = Array.deleteValue(myArray, NaN);

Array.show(myArray);

selectWindow("myArray");

saveAs("Results", savedir + "C" + j + "_C" + k + ".csv");

total = 0;

for (l=0; l<lengthOf(NoNaN); l++) {

total=total+NoNaN[l];

}

norm_cross_coeff[i-1] = total / root_squared_sums[j-1] / root_squared_sums[k-1];

i=i + 1;

Array.show(norm_cross_coeff);

close("Result of C" + j);

close("Results");

close("C" + j + "_C" + k + ".csv");

}

norm_cross_coeff[i-1] = "column" + j + 1;

i=i+1;

}

selectWindow("norm_cross_coeff");

saveAs("Results", savedir + "norm_cross_coeff.csv");

selectWindow("root_squared_sums");

saveAs("Results", savedir + "root_squared_sums.csv");

run("Close All");

**References**

1 Schindelin, J. *et al.* Fiji: an open-source platform for biological-image analysis. *Nat Methods* **9**, 676-682, doi:10.1038/nmeth.2019 (2012).

2 Arganda-Carreras, I. *et al.* Trainable Weka Segmentation: a machine learning tool for microscopy pixel classification. *Bioinformatics* **33**, 2424-2426, doi:10.1093/bioinformatics/btx180 (2017).

3 Sternberg, S. R. Biomedical Image-Processing. *Computer* **16**, 22-34 (1983).
